# LPIH2V: LncRNA-protein interactions prediction using HIN2Vec based on heterogeneous networks model

**DOI:** 10.3389/fgene.2023.1122909

**Published:** 2023-02-10

**Authors:** Meng-Meng Wei, Chang-Qing Yu, Li-Ping Li, Zhu-Hong You, Zhong-Hao Ren, Yong-Jian Guan, Xin-Fei Wang, Yue-Chao Li

**Affiliations:** ^1^ School of Information Engineering, Xijing University, Xi’an, China; ^2^ College of Grassland and Environment Sciences, Xinjiang Agricultural University, Urumqi, China; ^3^ School of Computer Science, Northwestern Polytechnical University, Xi’an, China; ^4^ Xijing University, Xi’an, China

**Keywords:** lncRNA-protein interaction, heterogeneous information network, network embedding, HIN2Vec, behavioral features

## Abstract

LncRNA-protein interaction plays an important role in the development and treatment of many human diseases. As the experimental approaches to determine lncRNA–protein interactions are expensive and time-consuming, considering that there are few calculation methods, therefore, it is urgent to develop efficient and accurate methods to predict lncRNA-protein interactions. In this work, a model for heterogeneous network embedding based on meta-path, namely LPIH2V, is proposed. The heterogeneous network is composed of lncRNA similarity networks, protein similarity networks, and known lncRNA-protein interaction networks. The behavioral features are extracted in a heterogeneous network using the HIN2Vec method of network embedding. The results showed that LPIH2V obtains an AUC of 0.97 and ACC of 0.95 in the 5-fold cross-validation test. The model successfully showed superiority and good generalization ability. Compared to other models, LPIH2V not only extracts attribute characteristics by similarity, but also acquires behavior properties by meta-path wandering in heterogeneous networks. LPIH2V would be beneficial in forecasting interactions between lncRNA and protein.

## 1 Introduction

LncRNAs are a group of RNA molecules that are transcribed and do not have the competence to code for proteins. LncRNAs are normally defined as RNAs over 200 nucleotides long with no potential for symbolic coding, often playing administrative roles in the regulation of gene expression (Prensner and Chinnaiyan, 2011; Volders et al., 2013). LncRNAs can act as gene controllers and are involved in many of the biological processes as other epigenetic mechanisms (Esteller, 2011). LncRNA is poorly identified in the genome and is known as the “dark matter” of the genome. The function of most ncRNAs remains unclear, and a lot of lncRNAs may have no apparent function. In recent decades, a growing number of studies have uncovered that lncRNA plays an influential role in many biological processes (Wang et al., 2018). With ongoing developments in deep RNA sequencing and advanced epigenomics technologies, the rate of discovery of new lncRNA genes is quickly outstripping the rate at which they can be described. When lncRNAs are out of order, they may induce a variety of diseases, such as Alzheimer (Ng et al., 2013), autism (Cook and Scherer, 2008), cardiovascular diseases (Congrains et al., 2012), and cancer (Chen et al., 2017; Rathinasamy and Velmurugan, 2018). For instance, genetic deletion of the lncRNA locus on human chromosome 2 leads to serious congenital limb deformities (Allou et al., 2021). LncRNAs play regulatory roles in immune response in prostate cancer (Hu et al., 2021). PlncRNA-1 significantly increased in prostate cancer cells (Cui et al., 2013). ANRIL is obviously associated with coronary heart disease, type 2 diabetes and many types of cancer (Pasmant et al., 2011). A new lncRNA, FASRL, was recently discovered to boost the proliferation of hepatocellular carcinoma (HCC) cells *in vitro* and *in vivo* (Peng et al., 2022).

In recent years, probing the interactions between lncRNAs and proteins has been one of the primary ways to deduce the functions of lncRNAs and to do further research on lncRNAs. LncRNA-protein interactions (LPIs) throughout their lifetimes, regulating not only maturation, nuclear export, stability, and eventually translation, but also the functions of RNAs. Given the critical role of lncRNA in various biological processes and complex diseases, there is an urgent need to uncover potential lncRNA-protein associations. Hence, to effectively predict LPIs, a variety of methods have recently been proposed, classified into two broad categories, including experimental and computational methods (Zhang and Fan, 2017; Li et al., 2022). Given the number and variety of lncRNA and protein, an exhaustive experimental validation of each lncRNA and protein would be impractical (Alipanahi et al., 2015). Therefore the need for the use of computational methods to screen for potential LPIs in these high-throughput assays and then validate them experimentally. Current computational methods fall into two broad categories: network-based and machine-learning methods.

Nowadays, several computational methods for predicting LPIs have been proposed. Muppirala et al. (2011) proposed RPISeq, a family of classifiers for predicting RNA-protein interactions, using only sequence information. This model extracts features from lncRNA and protein sequences using two classifiers, support vector machine (SVM) and random forest (RF), respectively, and the results show that the SVM classifier predicts more accurately. Wang et al. (2013) proposed the model, which is based on a Bayesian classifier that first collects a set of known RNA-protein interactions as positive criteria and extracts sequence-based features to represent each RNA-protein pair, selecting valid features by reducing the likelihood ratio score. These valid features are used to build an extended Bayesian classifier for training RNA-protein interaction prediction. Lu et al. (2013) transformed the sequences of amino acids into numerical feature vectors. This model which is named lncPro transformed the sequence information of lncRNAs and proteins into feature vectors then reduced the dimensionality of the lncRNA and protein feature vectors using Fourier series, and finally integrated the information of both using matrix multiplication to score each lncRNA-protein pair. A new sequence distributed representation learning-based method for potential LPI prediction, named LPI-Pred, was developed by Yi et al. (2020a) which regarded lncRNA and protein sequences as “words” in natural language processing and trained the RNA2vec and Pro2vec models using word2vec. However, the above approach only considers information about the properties of lncRNAs and proteins and not their behavior, which has now been demonstrated in many articles in the field of bioinformatics to be powerful in improving the prediction of LPIs (Guan et al., 2022; Guo et al., 2022; Ren et al., 2022). Ge et al. (2016) which proposed and tested a new computational approach, LPBNI, which constructs a bipartite network of lncRNA-proteins, using information about LPIs to link lncRNAs and proteins if they are known to interact with each other. Yang et al. (2016) proposed a model which represented and analyzed the interactions between lncRNAs and proteins as a heterogeneous network. The model used a correlation-search algorithm called HeteSim to predict lncRNA interactions with proteins. Deng et al. (2018) proposed a model, PLIPCOM, which constructed a network from known sequence similarities and LPIs information. First, the model used the random walk algorithm to extract diffusion features and HeteSim features, then reduced the dimensionality by the SVD algorithm, respectively. While the above approach takes into account the acquisition of behavioral information through network embedding, it does not take into account the role of meta-paths in heterogeneous networks.

In this article, a novel model for predicting LPIs based on heterogeneous network embedding, LPIH2V, was proposed. Potential vectors of nodes in heterogeneous networks are learned and represented using a neural network-based HIN2Vec (Fu et al., 2017). The extracted feature vectors are then used to predict LPIs using an SVM classifier. After the 5-fold cross-validation test, the results show that LPIH2V has high accuracy and stability. At the same time, the LPIH2V model achieved the best prediction accuracy compared to known models for the same dataset.

## 2 Results and discussion

### 2.1 Evaluation criteria

In this experiment, we evaluated the performance of LPIH2V using commonly adopted metrics and the 5-fold cross-validation method (Yu et al., 2022). The dataset is divided into five equal subgroups, with data from each subset used for testing in turn and data from the remaining four subsets used for training data. We repeated this process to ensure that each part served as the test set. The mean of the five predictions was ultimately used as the final evaluation result. Six metrics were used to evaluate LPIH2V performance: accuracy, precision, recall, F1 score, SPE, and MCC. For the evaluation criteria above, higher values represent better performance and better performance.

A common description of the ACC is the systematic error, which indicates the discrepancy between the predicted result and the true value. PRE refers to the proportion of true positives in statistical and diagnostic testing. REC measures the proportion of correct positive identifications, also refers to as sensitivity. F1 scores represent the summed mean of accuracy and sensitivity. SPEC is the probability of a negative test result. MCC is the correlation coefficient between true and predicted values. TP and TN are the numbers of correctly identified positive and negative samples, respectively. FN and FP are the numbers of incorrectly identified positive and negative samples, respectively. These metrics can be defined as:

$$ACC=\frac{TP+TN}{TP+TN+FP+FN}$$

$$PRE=\frac{TP}{TP+FP}$$

$$REC=\frac{TP}{TP+FN}$$

$$F1=\frac{2\times PRE\times REC}{PRE+REC}$$

$$SPEC=\frac{TN}{FP+TN}$$

$$MCC=\frac{TP\times TN-FP\times FN}{\sqrt{\left(TP+FP\right)\left(TP+FN\right)\left(TN+FP\right)\left(TN+FN\right)}}$$

In addition to the above metrics, we also used AUC, the area under the ROC curve, to evaluate our model. We used the average of the five results to ensure the accuracy of the prediction results.

### 2.2 Evaluation of predictive capability

**FIGURE 1:**
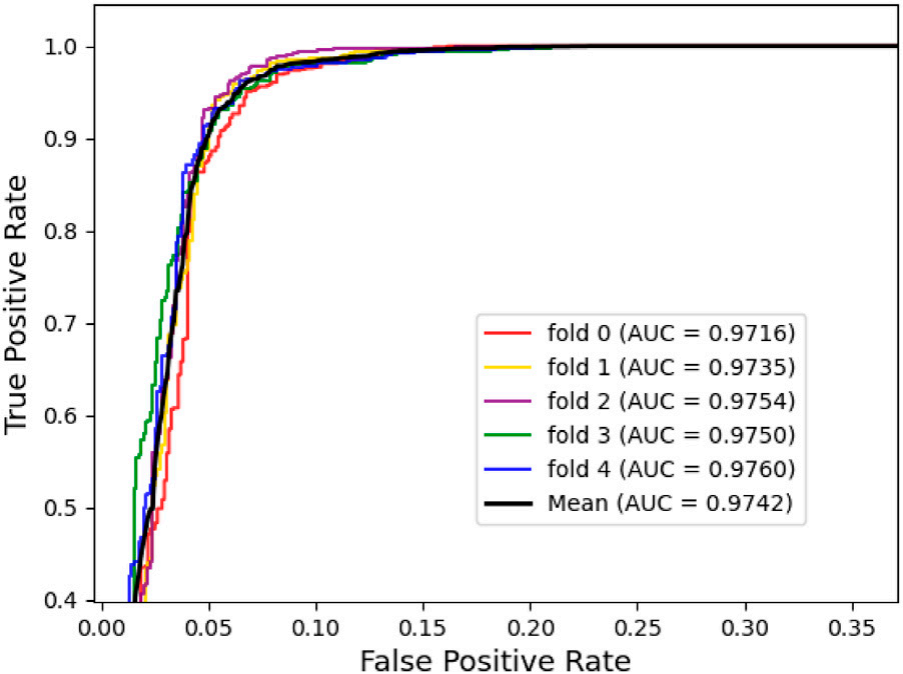
ROC curves plot for LPIH2V.

**FIGURE 2:**
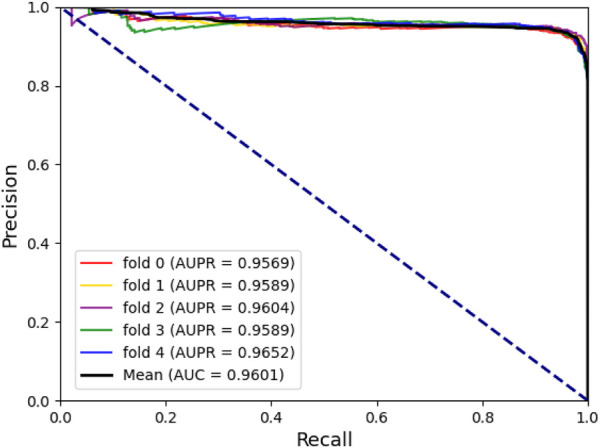
PRE curves plot for LPIH2V.

To assess the predictive power of the model, we used the 5-fold cross-validation and plotted the ROC and PRE curves for the 5 trials, as shown in Figure 1 and Figure 2. The mean predictive AUC for LPIH2V was 0.974. In addition, Table 1 summarizes the results of the model under 5-fold cross-validation.

**TABLE 1:** 5-fold cross-validation results for LPIH2V.

Test set	PRE(%)	REC (%)	SPE (%)	ACC(%)	MCC(%)	F1 (%)	AUC(%)
1	94.64	98.47	94.43	96.45	92.97	96.52	97.90
2	91.94	98.47	91.37	94.92	90.06	95.09	97.40
3	93.33	97.92	93.01	95.46	91.04	95.57	97.10
4	93.13	97.81	92.79	95.30	90.72	95.42	97.90
5	92.75	97.81	92.35	95.08	90.30	95.21	97.50
Average	93.16 ± 0.88	98.10 ± 0.31	92.79 ± 0.99	95.44 ± 0.54	91.02 ± 1.03	95.56 ± 0.51	97.56 ± 0.31

### 2.3 Comparison with graph embedding methods

**FIGURE 3:**
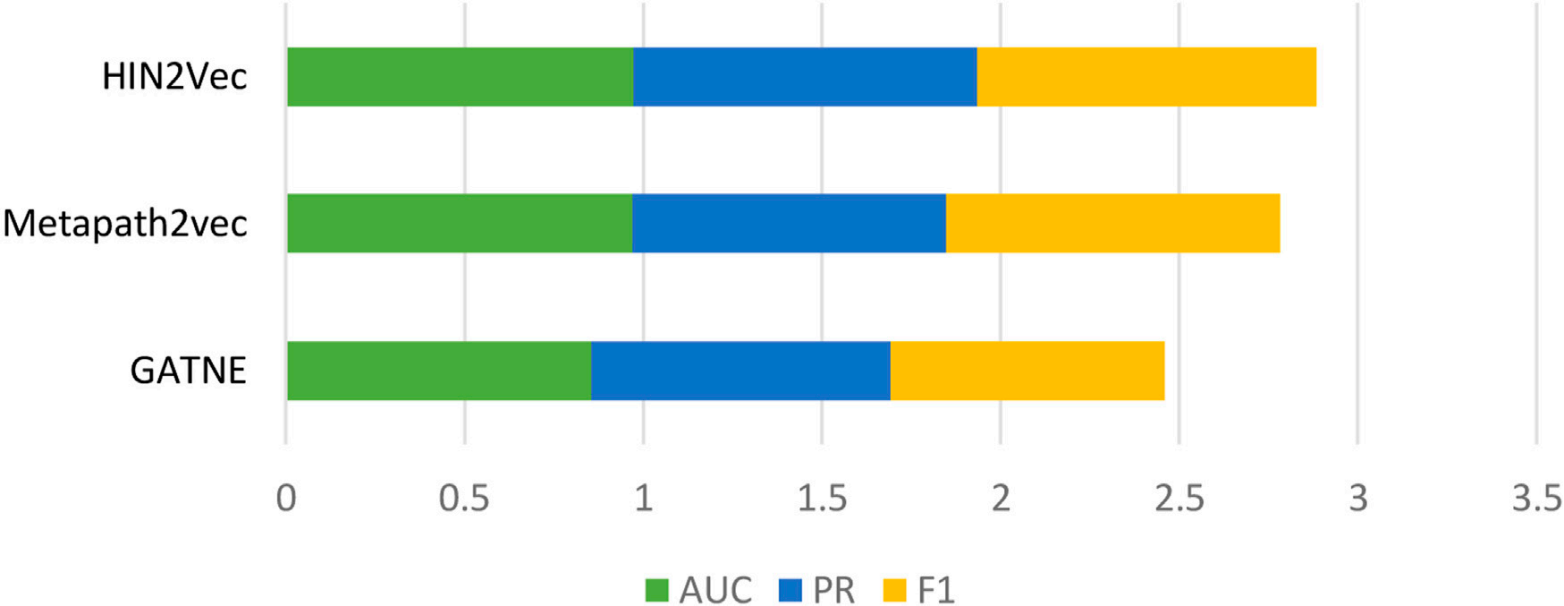
Graph embedding methods comparison.

Behavioral attributes are a very important feature. To demonstrate the predictive power of our model, we compared three heterogeneous network graph embedding methods based on meta-path theory (Yi et al., 2022). GATNE uses base embedding and edge embedding to extract hidden properties of different types of edges between nodes. Metapath2vec travels through the network through custom meta-paths to capture potential behavioral features. HIN2Vec computes meta-paths in heterogeneous networks, then travels across the calculated meta-paths to capture properties of each node across the board. In the same dataset, as shown in Figure 3, the results show that the HIN2Vec method used in our model is effective in predicting LPIs.

### 2.4 Comparing with different base classifiers

**FIGURE 4:**
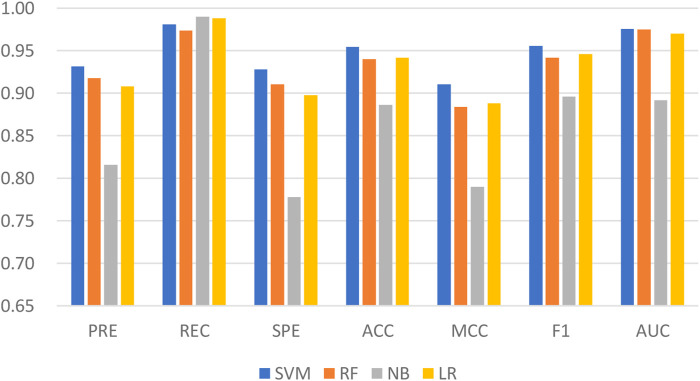
Comparison of the performance of LPIH2V and other classifiers.

Machine learning has been applied successfully to predict LPIs. In the LPIH2V, we used the SVM to classify the integrated features. SVM is one of the well-known algorithms based on statistical learning theory. In order to fully demonstrate the superiority of fusing information from attribute features and behavioral features, four classical machine learning algorithms were tested on LPIH2V, including SVM, RF, Gaussian NB (NB), and Logistic Regression (LR). As we can see in Figure 4, the proposed model achieved the best results according to precision, recall, SPE, accuracy, MCC, F1, and AUC. These results indicate that the SVM classifier is a good fit for the proposed model and can predict LPIs effectively.

### 2.5 Comparison with other models

In addition, to verify the validity and stability of LPIH2V, we compared LPIH2V with other computational methods include CF (Sarwar et al., 2001), RWR (Köhler et al., 2008), LPBNI (Ge et al., 2016), SFPEL-LPI (Zhang et al., 2018a), LPIHN (Li et al., 2015), LPLNP (Zhang et al., 2018b), RPI-SE (Yi et al., 2020b), IPMiner (Pan et al., 2016), and LncPNet (Zhao et al., 2021a). The CF model uses cosine similarity and Pearson correlation similarity to calculate the correlation between the two users based on collaborative filtering recommendation algorithms. To define the similarity of nodes in networks, the RWR model uses random walk with restart to calculate the distance between nodes in the network. LPBNI used information on known RNA-protein interactions to build a lncRNA-protein bipartite network. Following this, a propagation method was performed in the bipartite network to score and rank the candidate proteins for each lncRNA. The SFPEL-LPI model extracts lncRNA and protein sequence features using linear neighborhood similarity, then predicts LPIs using the feature projection ensemble learning method. The LPIHN model constructs a heterogeneous network by linking the lncRNA-lncRNA similarity network, protein-protein interaction network, and lncRNA-protein interaction network. LncRNAs and proteins were scored in heterogeneous networks using random walk with restart. LPLNP calculates linear neighborhood similarities in feature spaces and transfers them to interaction space to predict unknown interactions through the label propagation process. RPI-SE integrates three independent models, including XGBoost, SVM, and ExtraTree, to predict ncRNA-protein interactions using sequence information and fully exploit the potent properties of RNA and protein employing PWM and K-mer sparse matrices. The IPMiner model extracts the 3-mers and 4-mers from protein and RNA sequences, respectively, and then uses a stacked autoencoder to derive high-level features from the retrieved RNA and protein sequence features, respectively. The above stacked autoencoders were fine-tuning using the label information from the training data to change the index of the network. The LncPNet model constructs a heterogeneous network by calculating the similarity between lncRNA-lncRNA and protein-protein, then extracts features using metapath2vec. Table 2 shows that our model achieved the highest AUC value of 0.976. LPIH2V performed the best on other metrics because we used the HIN2Vec method to extract features that capture rich relational semantics and details of the network structure to learn the representation of nodes in HIN. In addition, the model learns the representation of meta-paths for meta-path analysis.

**TABLE 2:** Performance comparison of LPIH2V with other tools for predicting lncRNA-protein interactions.

Method	PRE	REC	SPE	ACC	MCC	F1	AUC
CF	0.583	0.894	0.361	0.627	0.301	0.706	0.761
RWR	0.739	0.798	0.717	0.757	0.517	0.767	0.830
LPBNI	0.740	0.840	0.698	0.769	0.548	0.785	0.859
SFPEL-LPI	0.769	0.920	0.724	0.822	0.657	0.838	0.916
LPIHN	0.807	0.966	0.769	0.867	0.750	0.879	0.938
LPLNP	0.832	0.943	0.810	0.876	0.761	0.884	0.944
RPI-SE	0.877	0.974	0.863	0.919	0.843	0.923	0.959
IPMiner	0.886	0.970	0.875	0.922	0.849	0.926	0.961
LncPNet	0.908	0.957	0.903	0.930	0.860	0.932	0.971
LPIH2V	0.932	0.981	0.928	0.954	0.9102	0.956	0.976

## 3 Materials and methods

### 3.1 Datasets

In this experiment, lncRNA sequence data were obtained from NONCODE (Zhao et al., 2021b), protein sequence data from UniProt (UniProt Consortium, 2021), and known lncRNA-protein interaction data from NPInter (Teng et al., 2020). UniProt provides a complete compilation of all known protein sequence data and links it to a summary of validated experience or computational predictions of the protein’s functional information. To avoid data redundancy, the UniProt database consolidated the same protein stored in different databases into one and gives it a unique and specific identifier. The number of entries contained in UniProt had grown to over 65 million records. NPInter incorporated 600,000 new experimentally determined ncRNA interactions, primarily by hand mining the literature and processing high-throughput sequencing data to collect interaction data. Researchers integrated data from different sources and eliminated redundant entries. NONCODE was a comprehensive database of assembled and illustrated non-coding RNAs, typically animal lncRNAs. NONCODE not only provides access to the names and NONCODE IDs of commonly used lncRNAs, but some lncRNAs also support other database names for conducting searches. The number of lncRNAs has rapidly increased to 644,510, of which 173,112 were human lncRNAs. First, the LPIs information extracted from NPInter were limited to “Homo,” “lncRNA” and “protein” respectively, and filtered for operationally proven human LPIs information. The resulting lncRNA ID and protein ID were then plotted as NONCODE ID and UniProt IDs, respectively. Finally, invalid lncRNA and protein information was deleted from the sequence information, leaving 4,578 pairs of known LPIs information.

### 3.2 Overview of methods

**FIGURE 5:**
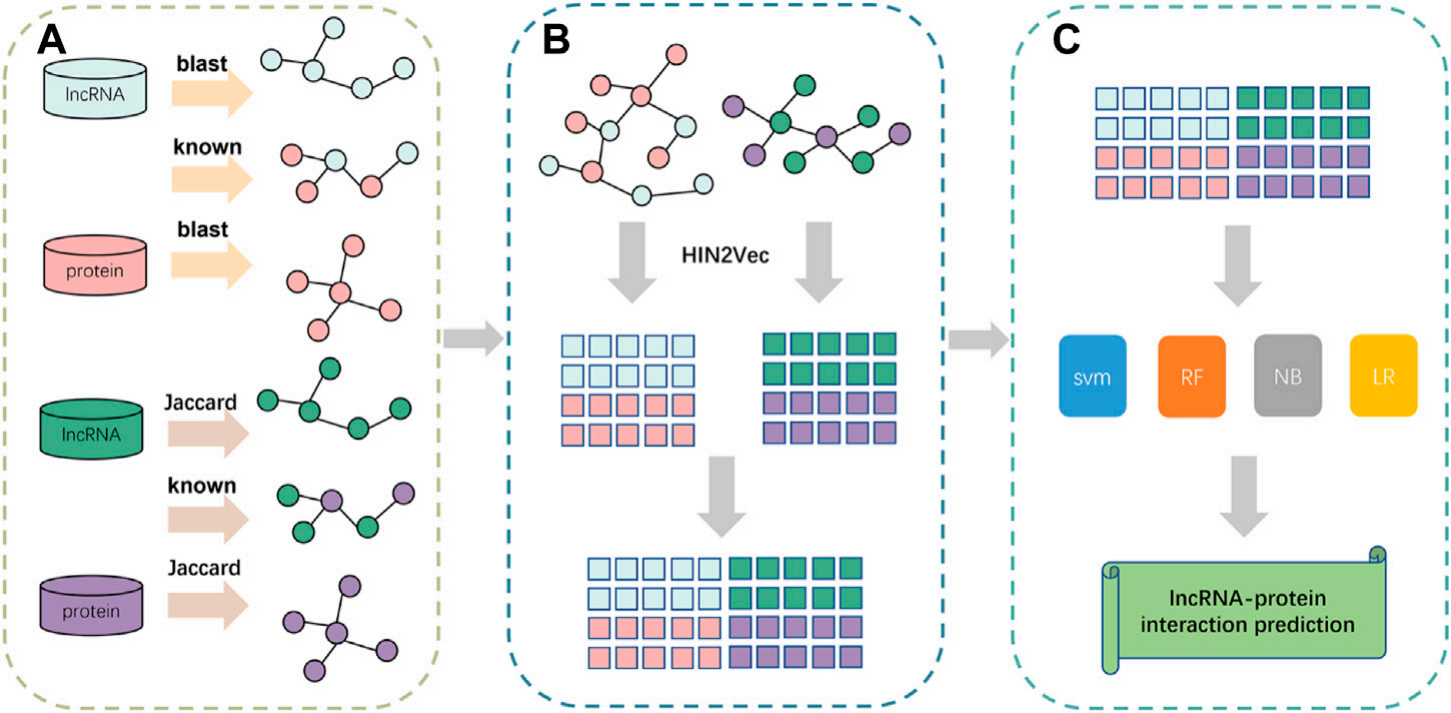
Pipeline of the framework of LPIH2V. **(A)** Calculation of similarity between lncRNA-lncRNA and protein-protein. **(B)** Construction of heterogeneous networks using similarity information obtained from **(A)** and extraction of node features using HIN2Vec. **(C)** Use of multiple classifiers to predict LPIs.

In this paper, we propose a prediction framework based on fusing attribute features and behavioral features, called LPIH2V. As shown in Figure 5, LPIH2V is divided into three parts. In the first part, we compute Jaccard similarity and BLAST similarity for the lncRNAs and proteins, respectively, given the lncRNA-lncRNA and protein-protein sequence information in the dataset, as well as the known LPIs information. In the second part, we construct two heterogeneous networks of lncRNA-protein and use the HIN2Vec method to extract behavioral features of the nodes in the network based on meta-paths. The features derived from the two heterogeneous networks were integrated. In the third part, we predict the LPIs by multiple classifiers. The feature vectors are fed into the classifiers to produce predictions. It is worth noting that many models ignore the semantic relationships between different node types. We fully learn the structural information of the nodes embedded in the network by constructing a heterogeneous network and HIN2Vec method. The feature information obtained through learning is fused to retain the sequence information of the nodes.

### 3.3 Sequence similarity calculation

The calculation of similarity is an important part of gene association prediction. Basic Local Alignment Search Tool (BLAST) (Fu et al., 2018) is a dual sequence local alignment algorithm proposed by Altschul et al., in 1990. As a set of analytical tools for similarity comparisons in protein databases or DNA databases, which contains several separate procedures. These programs are defined depending on the query and the database. For example, if the query is for nucleic acid and the query database is a nucleic acid sequence database, then the blastn program should be selected. The basic concept of BLAST is to increase the speed of comparison by generating fewer but better-quality enhancement points. In this paper, we performed BLAST to obtain the similarity between every two lncRNAs and every two proteins. Jaccard similarity (Lu et al., 2018) is a popular approximation measure used to calculate the similarity between two objects. Jaccard similarity can be used to find the similarity between two asymmetric binomial vectors or to find the similarity between two sets. The Jaccard coefficient is often used between sequence-order insensitive texts. The higher the value of the Jaccard coefficient, the higher the similarity of the sample. We calculated lncRNA-lncRNA similarity and protein-protein similarity by using Jaccard similarity principle. The Jaccard coefficient is defined as the size of the intersection of the sample set divided by the size of the merge set. For example, $L_{i}$ and $L_{j}$ are datasets of two lncRNAs. The Jaccard similarity between any two sets of lncRNAs is computed as follows:

$$J\left(L_{i},L_{j}\right)=\frac{\left|L_{i}\cap L_{j}\right|}{\left|L_{i}\cup L_{j}\right|}$$

Jacquard similarity of proteins is calculated in the same way as lncRNA.

### 3.4 Heterogeneous network construction

In recent years, the precise extraction of personalization factors from data has become increasingly difficult due to the rapid growth of Big Data. Heterogeneous information networks (HINs) can represent the attribute information and structure information completely, which not only assumes two nodes are related but also distinguishes between different relationships among nodes and retains more contextual information by learning the relationship vectors jointly. In detail, the training data is represented using a bipartite graph $=\left(m,n,r,j/b\right)$ , where m and n are two nodes in the same category, and the r is the link between two nodes. The Jaccard and BLAST values of the two nodes are indicated by j and b respectively. For lncRNA, samples with Jaccard similarity greater than 0.5 and BLAST similarity less than 0.001 were selected. On the protein side, samples with Jaccard similarity greater than 0 and BLAST similarity less than 0.01 were selected. The heterogeneous networks are made up of these two similarity networks and known LPIs.

### 3.5 Heterogenous network embedding

**FIGURE 6:**
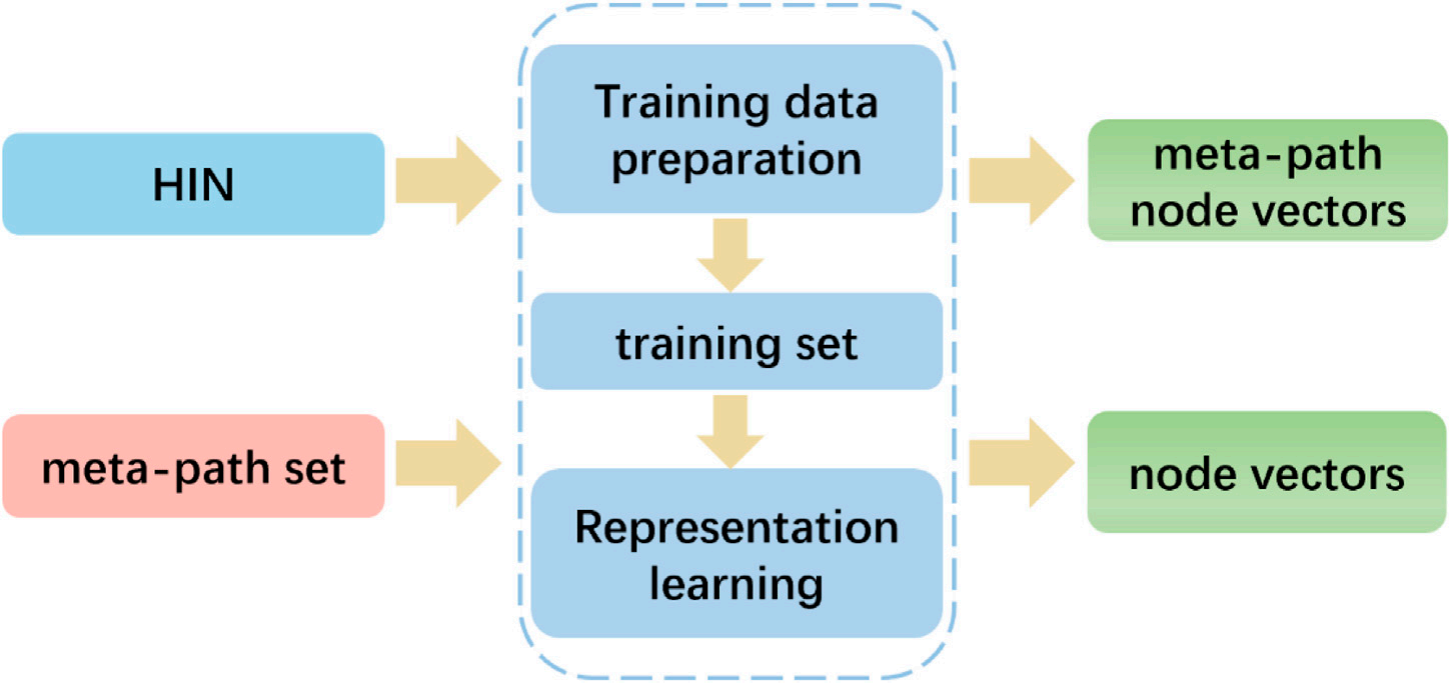
HIN2Vec flowchart: Extraction of node eigenvectors using HINs and meta-paths.

To capture the rich semantics embedded in heterogeneous networks by exploiting the different types of relationships among nodes, we employed the HIN2Vec method. Unlike metapath2vec, which follows a given meta-path pattern, HIN2Vec selects nodes randomly. In particular, as illustrated in Figure 6, the HIN2Vec framework consists of two phases: training data preparation and representation learning. The HIN2Vec matching target relation data is generated based on random wandering and negative sampling to represent the learning for the nodes in the network. To learn the node vectors and relationships between pairs of nodes, we maximized the likelihood of jointly predicting the relationships between lncRNAs and proteins.

**FIGURE 7:**
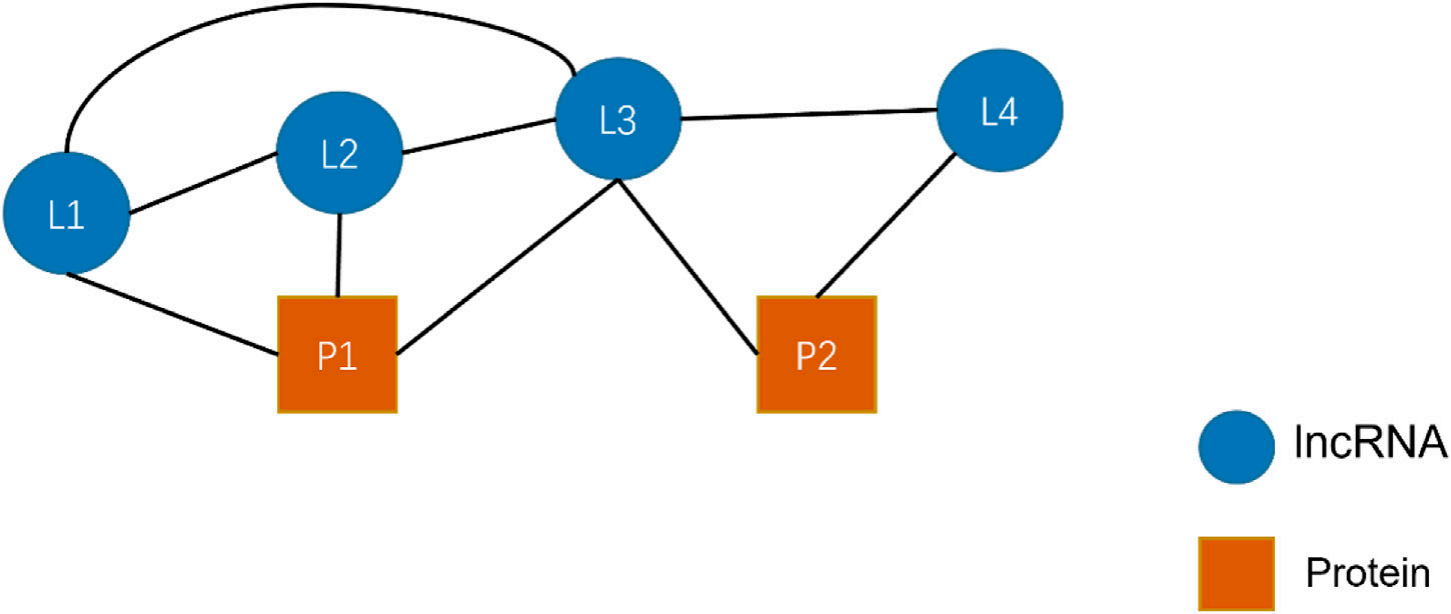
An example of a simple heterogeneous network of lncRNA-protein interactions.

Figure 7 shows a simple HIN consisting of four lncRNAs (L) L1, L2, L3, L4, and two proteins (P) P1 and P2. In this figure, edges represent the relationship that exists between nodes. We restricted the meta-path length to be at most 2. Giving R denotes a collection of targets that includes all relations between nodes. Thus, $R=\left\{L-L,L-P,P-L,L-L-L,L-L-P,L-P-L,P-L-L,P-L-P\right\}$ , L1 to P1 as $\left\{L-P,L-L-P\right\}$ , entering training data is $\left\{x:L_{1},y:P_{1},output:\left[0,1,0,0,1,0,0,0\right]\right\}$ . From the above example, it is straightforward to see that the HIN2Vec method traverses all the meta-paths in the heterogeneous network and then learns each potential feature vector.

**FIGURE 8:**
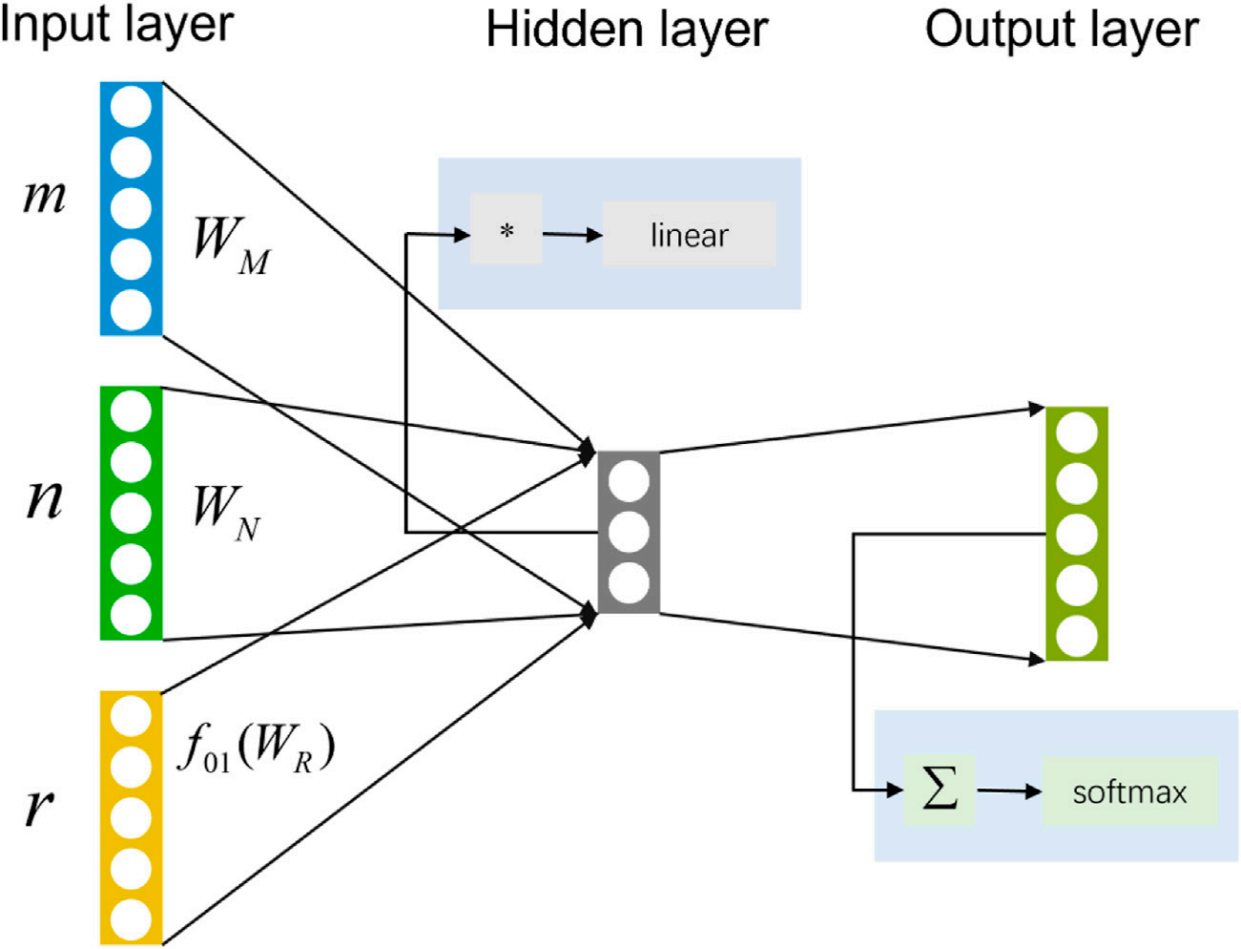
HIN2Vec NN model includes input layer, hidden layer and output layer.

To reduce the time required to traverse all the meta-paths, the HIN2Vec based on a neural network (HIN2Vec NN) will predict the probability of the relationship between two nodes in advance. A three-layer feedforward neural network model adopted by HIN2Vec as a binary classifier. As shown in Figure 8, input the two nodes m , n and the relationship r between m and n into the binary classifier. The three parameters at the input layer are mapped into three one-hot feature vectors $\vec{m}$ , $\vec{n}$ , $\vec{r}$ , which mapped into potential vectors $W_{M}^{\prime }\vec{m}$ , $W_{N}^{\prime }\vec{n}$ and $W_{R}^{\prime }\vec{r}$ . In the hidden layer, considering that the semantics of nodes and relations are different, and therefore their representation space should not be the same, which applied a regularization function $f_{01}\left(.\right)$ to limit the potential vector for r to be between 0 and 1, then use the Hadamard function and apply Identity function to activate the three vectors. The output layer uses the Summation function as input, summing up three values. To realize logistic classification, the Sigmoid function is used as the activation function.

HIN2Vec uses backpropagation training algorithms and stochastic gradient descent to optimize the model. Assuming a training set $D\left(m,n,r,L\left(m,n,r\right)\right)$ , which include m , n and the relationship r between m and n . We denote by L if there is any interaction between nodes m and n , expressed as 0 or 1. A maximization objective function O is used to adjust the weights of each parameter $W_{M}$ , $W_{N}$ and $W_{R}$ , and then maximize log⁡O . Given an objective function O and a derivation of log⁡O such that:

$$O\propto \log O=\Sigma_{m,n,r\in D}{\log O}_{m,n,r}\left(m,n,r\right)$$

In particular, for an input of training data $\left(m,n,r,L\left(m,n,r\right)\right)$ , when $L\left(m,n,r\right)$ is 1, $O_{m,n,r}\left(m,n,r\right)$ intends to maximize $P\left(\left.r\right|m,n\right)$ , Or else, $O_{m,n,r}\left(m,n,r\right)$ intends to minimize $P\left(\left.r\right|m,n\right)$ . Here $B\left(m,n,r\right)$ , a binary value, indicates whether m and n have r. Therefore, $O_{m,n,r}\left(m,n,r\right)$ , ${\log O}_{m,n,r}\left(m,n,r\right)$ and $P\left(\left.r\right|m,n\right)$ as follows:

$$O_{m,n,r}\left(m,n,r\right)=\left\{\begin{array}{c} P\left(\left.r\right|m,n\right)\;if\;L\left(m,n,r\right)=1\\ 1-P\left(\left.r\right|m,n\right)\;if\;L\left(m,n,r\right)=0 \end{array}\right.$$

$${\log O}_{m,n,r}\left(m,n,r\right)=B\left(m,n,r\right)\log P\left(\left.r\right|m,n\right)+\left[1-B\left(m,n,r\right)\right]\log\left[1-P\left(\left.r\right|m,n\right)\right]$$

$$P\left(\left.r\right|m,n\right)=sigmoid\left(\sum W_{M}^{\prime }\vec{m}⊙W_{N}^{\prime }\vec{n}⊙f_{01}\left(W_{R}^{\prime }\vec{r}\right)\right)$$

Finally, for each training data entry, the algorithm reverses weights in $W_{M}^{\prime }\vec{m}$ , $W_{N}^{\prime }\vec{n}$ and $W_{R}^{\prime }\vec{r}$ based on the gradients of ${\log O}_{m,n,r}\left(m,n,r\right)$ differentiating with respect to $W_{M}^{\prime }\vec{m}$ , $W_{N}^{\prime }\vec{n}$ and $W_{R}^{\prime }\vec{r}$ , respectively, as follows:

$$W_{M}^{\prime }\vec{m}:=W_{M}^{\prime }\vec{m}+\frac{d\log OF_{m,n,r}\left(m,n,r\right)}{dW_{M}^{\prime }\vec{m}}$$

$$W_{N}^{\prime }\vec{n}:=W_{N}^{\prime }\vec{n}+\frac{d\log OF_{m,n,r}\left(m,n,r\right)}{dW_{N}^{\prime }\vec{n}}$$

$$W_{R}^{\prime }\vec{r}:=W_{R}^{\prime }\vec{r}+\frac{d\log OF_{m,n,r}\left(m,n,r\right)}{dW_{R}^{\prime }\vec{r}}$$

## 4 Conclusion

With the rapid development of computer performance, traditional wet experiments have limitations compared to computational methods. The cost of experiments can be greatly reduced and experimental time saved. The computational method not only reduces the interference of other factors in the experiment but also provides specific ideas for biological experiments. Most lncRNAs exert their effects by interacting with proteins, so the prediction of LPIs is critical for the proper functioning of lncRNAs.

The purpose of the study was to propose a model for predicting lncRNA-protein interactions that combines sequence characteristics and behavioral properties to fully exploit node information, and we constructed heterogeneous networks using similarity principles. LPIH2V builds heterogeneous networks through the principle of sequence similarity. HIN2Vec is used to learn the behavioral features of the nodes in the network. This takes into account both the sequence characteristics and the behavioral characteristics of the nodes. We also used a sparse automatic encoder to upgrade the eigenvectors, converting vectors from 64 dimensions to 128 dimensions for comparison, and using different classifiers to predict vectors for elevation. The results show that the recall score of 0.98 using stochastic forest classifiers indicates that the model can identify positive and negative samples more accurately by raising the dimensions. To verify the robustness and reliability of the proposed method, we used multiple classifiers to compare predictions and found that SVM worked best. An additional 5-fold comparison test was performed to test the accuracy of the prediction model. Then used the best SVM classifier to compare with other advanced methods, and finally concluded that our approach has superiorities in predicting LPIs. Undoubtedly, our proposed model has the potential to provide a useful guide for biomedical research in LPIs prediction. With advances in technology, more efficient feature extraction strategies and incorporation of other information into the model could lead to greater accuracy and improved performance, such as disease and miRNA.

However, LPIH2V has some shortcomings. Learning node features using HIN2V can be time-consuming. The number of datasets also has an impact on the model results. The following will look for ways to optimize the learning time of the model and to further expand the number of datasets.

## Data Availability

The original contributions presented in the study are included in the article/supplementary materials, further inquiries can be directed to the corresponding author/s.
